# Supplementary material for: Typhoon damage on a shallow mesophotic reef in Okinawa, Japan
Source: PeerJ. 2013 Sep 3;1:e151. doi: 10.7717/peerj.151 (PMC3767277; doi:10.7717/peerj.151)
Supplement: Table S1 — Coral species and functional groups (documented at Ryugu Reef with percent coral cover and percent cover at Stations 2 (30 + m) and 3 (<30 m). Functional groups: 1, massive; 2, encrusting; 3, foliose; 4, columnar; 5, plate-like; 6, bushy; 7, arborescent; 8, unattached. [file peerj-01-151-s001.docx]

| **Species (functional group)** | **Station 2 before** | | **after** | | **change** | **Station 3 before** | | **after** | | **change** | |
| --- | --- | --- | --- | --- | --- | --- | --- | --- | --- | --- | --- |
| *Acropora echinata* (6) | 0 | | 0 | | - | 0.2 | | 0 | | 0.2 | |
| *Acropora* sp. 1 (6,7) | 1.9 | | 0 | | 1.9 | 2.7 | | 1.3 | | 1.4 | |
| *Acropora* sp. 2 (5,6) | 0 | | 0.2 | | -0.2 | 3.7 | | 0.4 | | 3.3 | |
| *Acropora* sp. 3 (6) | 0 | | 0 | | - | 0.8 | | 0.4 | | 0.4 | |
| *Acropora* sp. 4 (5,6) | 1.1 | | 0.2 | | 0.9 | 0.2 | | 0.1 | | 0.1 | |
| *Acropora* sp. 5 (6) | 0 | | 0 | | - | 0.4 | | 0 | | 0.4 | |
| *Acropora* sp. 6 (6) | 0 | | 0 | | - | 2.7 | | 0 | | 2.7 | |
| Agaraciidae sp. 1 (3) | 0 | | 0 | | - | 0.2 | | 0 | | 0.2 | |
| *Astreopora* sp.1 (1,2) | 0 | | 0 | | - | 0 | | 0 | | - | |
| *Australomussa* sp. 1 (1,2,3) | 0 | | 0 | | - | 0.2 | | 0.2 | | 0 | |
| *Caulastrea* sp. 1 (1) | 0 | | 0 | | - | 0.1 | | 0 | | 0.1 | |
| Coral rubble (N/A) | 4.0 | | 37.3 | | -33.3 | 37.9 | | 49.3 | | -11.4 | |
| *Ctenactis albitentaculata* (8) | 0 | | 0 | | - | 0 | | 0.1 | | -0.1 | |
| *Ctenactis crassa* (8) | 0 | | 0 | | - | 1.1 | | 1.2 | | -0.1 | |
| *Ctenactis echinata* (8) | 0.6 | | 0 | | 0.6 | 0.5 | | 1.0 | | -0.5 | |
| *Danafungia horrida* (8) | 0 | | 0 | | - | 0.8 | | 0.9 | | -0.1 | |
| *Danafungia scruposa* (8) | 0 | | 0.1 | | -0.1 | 1.8 | | 0.7 | | 1.1 | |
| *Dipsastraea* sp. 1 (1) | 0 | | 0 | | - | 0 | | 0.3 | | -0.3 | |
| *Echinophyllia orpheensis* (1) | 0 | | 0 | | - | 0.2 | | 0 | | 0.2 | |
| *Echinophyllia* sp. 1 (2,3) | 3.6 | | 0.4 | | 3.2 | 0 | | 3.5 | | -3.5 | |
| *Euphyllia glabrescens* (1) | 0 | | 0 | | - | 0.2 | | 0.3 | | -0.1 | |
| *Favites* sp. 1 (1) | 0 | | 0 | | - | 0 | | 0.3 | | -0.3 | |
| *Galaxea* sp. 1 (1,2,4) | 5.3 | | 1.8 | | 3.5 | 14.7 | | 9.0 | | 5.7 | |
| *Galaxea* sp. 2 (1,2) | 0 | | 0.1 | | -0.1 | 0.4 | | 0.7 | | -0.3 | |
| *Galaxea* sp. 3 (1,2) | 0 | | 0 | | - | 1.9 | | 0.6 | | 1.3 | |
| *Goniastrea* sp. 1 (1) | 0 | | 0 | | - | 0.1 | | 0 | | 0.1 | |
| *Halomitra pileus* (8) | 0 | | 0 | | - | 0.3 | | 0.2 | | 0.1 | |
| *Herpolitha limax* (8) | 0 | | 0 | | - | 0.7 | | 1.3 | | -0.6 | |
| *Leptoria* sp. 1 (1,2) | 0 | | 0 | | - | 0 | | 0 | | - | |
| *Lithophyllon concinna* (2,3) | 0 | | 0.4 | | -0.4 | 3.5 | | 4.8 | | -1.3 | |
| *Lithophyllon granulosa* (2,3) | 0 | | 0 | | - | 0 | | 0 | | - | |
| *Lithophyllon repanda* (2,3) | 7 | | 1.2 | | 5.8 | 8.9 | | 14.0 | | -5.1 | |
| *Lobophyllia* sp. 1 (1) | 0 | | 0 | | - | 0.1 | | 0.3 | | -0.2 | |
| Lobophyllidae sp. 1 (2,3) | 0 | | 0 | | - | 0.1 | | 0 | | 0.1 | |
| *Merulina* sp. 1 (2,3) | 0.1 | | 0 | | 0.1 | 0.3 | | 0 | | 0.3 | |
| *Millepora* sp. 1 (2) | 0 | | 0 | | - | 0.3 | | 0 | | 0.3 | |
| *Montipora* sp. 1 (2) | 0 | | 0 | | - | 0.2 | | 0 | | 0.2 | |
| *Montipora* sp. 2 (2,3) | 0 | | 0 | | - | 0 | | 0 | | - | |
| *Montipora* sp. 3 (2,3) | 0 | | 0 | | - | 0 | | 0 | | - | |
| *Montipora* sp. 4 (2,3) | 0 | | 0 | | - | 0.7 | | 0 | | 0.7 | |
| *Montipora* sp. 5 (2,3) | 0 | | 0 | | - | 0.5 | | 0 | | 0.5 | |
| *Montipora* sp. 6 (2,3) | 0 | | 0 | | - | 0.5 | | 0 | | 0.5 | |
| *Montipora* sp. 7 (2) | 0 | | 0 | | - | 0.1 | | 0 | | 0.1 | |
| *Mycedium elephantotus* (2,3) | 0 | | 0 | | - | 0.2 | | 0.3 | | -0.1 | |
| *Mycedium* sp. 1 (2,3) | 0 | | 0 | | - | 0.1 | | 0 | | 0.1 | |
| *Oxypora lacera* (2,3) | 0 | | 0 | | - | 0.3 | | 0 | | 0.3 | |
| *Pachyseris foliosa* (3) | 74.9 | | 56.6 | | 18.3 | 4.7 | | 2.7 | | 2.0 | |
| *Pachyseris rugosa* (3,5) | 0 | | 0 | | - | 0 | | 0.1 | | -0.1 | |
| *Pachyseris speciosa* (3,4) | 1.6 | | 1.1 | | 0.5 | 4.0 | | 0.3 | | 3.7 | |
| *Pavona cactus* (3) | 0 | | 0 | | - | 1.0 | | 3.7 | | -2.7 | |
| *Pectinia* sp. 1 (2,3) | | 0 | 0 | - | | 0.9 | 0 | | 0.9 | |  |
| *Plerogyra sinuosa* (1) | 0 | | 0 | | - | 0 | | 0 | | 0 | |
| *Pleuractis granulosa* (3) | 0 | | 0 | | - | 0 | | 0 | | 0 | |
| *Pleuractis paumotensis* (3) | 0 | | 0.5 | | -0.5 | 0.9 | | 1.4 | | -0.5 | |
| *Porites* sp. 1 (6) | 0 | | 0 | | - | 0.8 | | 0.1 | | 0.7 | |
| *Porites* sp. 2 (2) | 0 | | 0 | | - | 0 | | 0 | | - | |
| *Sandalolitha robusta* (3) | 0 | | 0 | | - | 0 | | 0.1 | | -0.1 | |
| *Seriatopora* sp. 1 (6) | 0 | | 0 | | - | 0.1 | | 0 | | 0.1 | |
| *Stylophora* sp. 1 (6) | 0 | | 0 | | - | 0.2 | | 0 | | 0.2 | |
| ? (N/A) | 0 | | 0 | | - | 0 | | 0.2 | | -0.2 | |
| Turbinaria sp. (N/A) | 0 | | 0 | | - | 0 | | 0.5 | | -0.5 | |
| Tridacninae(shell) (N/A) | 0 | | 0 | | - | 0 | | 0.3 | | -0.3 | |
